# The V-BRCH Project: building clinical trial research capacity for HIV and noncommunicable diseases in Nigeria

**DOI:** 10.1186/s12961-020-00656-z

**Published:** 2021-03-10

**Authors:** Muktar H. Aliyu, Mahmoud U. Sani, Donna J. Ingles, Fatimah I. Tsiga-Ahmed, Baba M. Musa, Carolyn M. Audet, C. William Wester

**Affiliations:** 1 Vanderbilt Institute for Global Health, Vanderbilt University Medical Center, 2525 West End Avenue, Suite 725, Nashville, TN 37203 United States of America; 2 Department of Health Policy, Vanderbilt University Medical Center, Nashville, TN United States of America; 3 Department of Medicine, Vanderbilt University Medical Center, Nashville, TN United States of America; 4 Department of Medicine, Bayero University and Aminu Kano Teaching Hospital, Kano, Nigeria; 5 Department of Community Medicine, Bayero University and Aminu Kano Teaching Hospital, Kano, Nigeria; 6 Africa Center of Excellence for Population Health and Policy, Bayero University, Kano, Nigeria; 7 Division of Infectious Diseases, Vanderbilt University Medical Center, Nashville, TN United States of America

**Keywords:** HIV, Noncommunicable diseases, Clinical trials, Research capacity, Training

## Abstract

Antiretroviral therapy has turned HIV into a chronic condition, with morbidity from HIV-associated noncommunicable diseases (NCDs) becoming more common as HIV-infected individuals live longer. In Nigeria, the additional challenge of an under-capacitated health system highlights the need for skilled clinical investigators who can generate evidence to tackle the double burden of HIV and NCDs. The Vanderbilt-Nigeria Building Research Capacity in HIV and Non-communicable Diseases (V-BRCH) programme is a training platform to create a cohort of skilled Nigerian investigators with the capacity to lead independent clinical trial research focused on the intersection of HIV and NCDs. V-BRCH will solidify an atmosphere of continuous mentoring and skills acquisition for physician faculty at the Aminu Kano Teaching Hospital via short- and medium-term learning opportunities, paired mentoring arrangements, and mentored research projects. Trainees will attend an annual faculty enrichment programme in Nashville, in addition to on-site workshops in Nigeria on HIV-associated NCD epidemiology, clinical trials methodology, evidence synthesis, qualitative research methods, stakeholder engagement, knowledge translation, and grant writing. Research-oriented junior faculty will undergo focused training in clinical trials administration and regulatory oversight. Scholars will share best practices through mentoring panels, regular ‘Works in Progress’ meetings, and monthly career development seminars. Competitive seed grants will be provided to mentor–mentee teams to promote targeted in-country pilot studies focused on HIV-associated NCDs. For long-term training, physician scientists will be supported to undergo enhanced Master of Public Health (MPH) training at Bayero University in Nigeria and Master of Science in Clinical Investigation (MSCI) training at Vanderbilt. Short-term regional courses, staff development workshops, and MPH curriculum refinement will help to strengthen institutional capacity in HIV-associated NCD clinical trial research. V-BRCH will create a cohort of skilled Nigerian scientists who will be able to compete for independent funding and design and implement high quality research that will generate evidence to inform policy and practice and lead to improved outcomes for Nigerians impacted by HIV-associated NCDs.

Many Africans are now living full lives with HIV, but at the cost of negative health outcomes associated with noncommunicable diseases (NCDs) [1–5]. These NCDs (e.g., cardiovascular disease, kidney disease, diabetes mellitus, pulmonary disease, neurologic disease, and others) increase with age and are often related to HIV, its treatment, and other host factors [6–11]. Research into this important area has been largely based on data from high-income countries [10]. Improving the treatment and prevention of NCDs in high HIV-burden resource-constrained settings like Nigeria will require region-specific evidence [12]. HIV infection in Nigerians is associated with higher triglycerides and lower high-density lipoprotein (HDL) cholesterol levels [13]. Hypertension, dyslipidemia, and metabolic syndrome are also more prevalent in antiretroviral therapy (ART)-exposed Nigerians compared to age- and sex-matched ART-naïve patients [14, 15]. This additional burden of NCDs within an already under-capacitated health system emphasizes the need to develop research capacity that can generate the evidence base to tackle the intersecting burden of HIV and NCDs in Nigeria.

Aminu Kano Teaching Hospital (AKTH, Kano, Nigeria) and Vanderbilt Institute for Global Health (Nashville, TN, USA) have a strong track record of collaborative research in Nigeria (Table 1). In 2008, Vanderbilt received funding from the Centers for Disease Control and Prevention (CDC) to implement a comprehensive HIV care and treatment programme. Multiple National Institutes of Health (NIH) grants have supported subsequent projects. In 2011, the Vanderbilt-AKTH team initiated the first NIH-funded randomized controlled trial (RCT) in Kano, Nigeria, to evaluate the feasibility of hydroxyurea for stroke prevention in sickle cell disease (SCD) [16, 17]. This pilot trial was followed by a larger phase III RCT and a Thrasher Foundation award to examine the effects of hydroxyurea in children with SCD who had already had a stroke [18]. In 2018, NIH funding was obtained to explore the efficacy of lisinopril for prevention of kidney disease in Nigerians with HIV [19]. Other NIH-funded NCD-relevant studies at AKTH include a project for task-shifting of epilepsy care to community health workers [20] and a feasibility trial to test the utility of reverse transcriptase oligonucleotide ligation assay-polymerase chain reaction (RT-OLA-PCR) assays for ART resistance testing in pregnant Nigerians. The Vanderbilt Building Research Capacity in HIV and Non-communicable Diseases (V-BRCH) program will build on this momentum of ongoing clinical trials to enhance research capacity at AKTH to conduct scientifically rigorous and ethically appropriate clinical trials, with a focus on the intersecting domains of HIV and NCDs.

**Table 1:** Examples of VIGH research and service initiatives in Nigeria

Study/Grant #	Focus	Years	Fund
1. Renal Risk Reduction ‘R3’ Trial 1U01DK112271 [20]	Chronic kidney disease prevention in HIV	2017–2022	NIDDK
2. SPRING Trial R01NS094041	Primary stroke prevention in sickle cell disease	2015–2020	NINDS
3. BRIDGE Trial R01NS113171	Childhood epilepsy	2019–2024	NINDS/FIC
4. RT-OLA-PCR assays for ARV resistance testing in Nigeria R21AI150828	HIV drug resistance testing in antenatal care	2019–2021	NIAID
5. BRIDGE Trial R21TW010899 [21]	Childhood epilepsy	2017–2019	NINDS/FIC
6. SPRINT Trial TRF1263028 [19]	Secondary stroke prevention in sickle cell disease	2016–2019	Thrasher Foundation
7. Optimizing PMTCT R01HD075075	Implementation science, PMTCT	2012–2016	NICHD/FIC
8. SPIN Trial, R21NS080639 [17, 18]	Primary stroke prevention in sickle cell disease	2012–2015	NINDS
9. FGHiN Nigeria, U2GGH00922	HIV service delivery	2013–2018	CDC/GAP
10. FGH Nigeria U2GPS001063	HIV service delivery	2008–2013	CDC/GAP

*ARV*  antiretrovirals, *CDC/GAP*  Centers for Disease Control and Prevention/Global AIDS Program, *FGHiN* Friends for Global Health Initiative in Nigeria; *FGH*  Friends in Global Health, *FIC*  Fogarty International Center, *SCD*  sickle cell disease, *NIAID*  National Institute for Allergy and Infectious Diseases, *NIDDK* National Institute of Diabetes and Digestive and Kidney Diseases, *NICHD* Eunice Kennedy Shriver National Institute of Child Health and Human Development, *NINDS*  National Institute of Neurological Disorders and Stroke, *PMTCT*  Prevention of mother-to-child HIV transmission

## Needs assessment

In 2015, the Nigeria Implementation Science Alliance (NISA) was established as a collaborative consortium of 20 Nigerian non-governmental organizations and academic and government institutions to build research capacity and generate implementation research evidence to address Nigeria’s major health problems. In collaboration with NIH, NISA has convened annual conferences in Nigeria. Post-conference surveys have highlighted Nigeria’s weak research base, inadequate clinical trial training opportunities, and inability of trainees to launch independent investigator careers due to lack of mentoring and poor infrastructural support [21–23].

Specific to AKTH, following the establishment of the World Bank-funded Africa Centre of Excellence in Population Health and Policy at Bayero University (Kano, Nigeria), a 2-day workshop was convened and attended by University leadership, research staff, community stakeholders, and international collaborators. An HIV Working Group during the workshop identified priority needs, including defining the burden of NCDs in Nigeria, elucidating the pathogeneses of NCDs in HIV, and developing cost-effective strategies to address the evolving NCD epidemics in Nigeria, among others. Skilled Nigerian scientists are needed to design and conduct high-quality clinical trials in these HIV-related NCD priority areas. A well-designed and responsive approach to research capacity-building will enable Nigeria to catch up with more resourced countries in the ability to conduct impactful HIV-NCD research.

## Institutional environment

Vanderbilt University School of Medicine (VUSM) has continued to attract the most accomplished and talented students. VUSM ranked 17th among 185 medical schools in the *U.S. News & World Report* 2019 survey of the top U.S. medical schools for research. Biomedical research at Vanderbilt is recognized for its contributions to the advancement of medicine.

- *The Master of Science in Clinical Investigation (MSCI) programme at Vanderbilt* trains investigators in the techniques of patient-oriented research. Trainees receive mentored training in clinical investigation and acquire a strong foundation in biostatistics, biomedical ethics, clinical pharmacology, human genetics, and assay methodology. Nearly 90% of MSCI alumni pursue careers in academic medicine.
- *Vanderbilt Institute for Global Health (VIGH)* was established in 2005 to facilitate the expansion and coordination of global health research, service, and training initiatives. VIGH engagements span the globe, including Africa, Central and South America, and Southeast Asia. Most participants in VIGH programmes return to their home countries and assume leadership positions (Table 2).
  *AKTH* was established in 1988 as a 500-bed tertiary-care facility affiliated with Bayero University and located in Kano, Nigeria. The HIV clinic at AKTH is one of the largest in the nation, with over 10,000 patients enrolled in active care.
- *Bayero University* (BUK) is the academic home for clinical and basic science programmes at AKTH. The College of Health Science comprises four faculties. The Faculty of Clinical Sciences hosts the Masters in Public Health (MPH) programme, in addition to the Community Health Officers training programme, providing opportunities for hands-on training in service delivery and research.
- *The Masters in Public Health (MPH) programme at BUK* was established in 2012, initially for physicians pursuing postgraduate fellowships, and has graduated 37 students. In 2018, the programme was opened to other health professionals, and the average class size was increased from 15 to 22 students per year. There is currently no clinical trials-focused course in the programme.

**Table 2:** Number of supported trainees by representative VIGH (VIGH) institutional training grants

Grant	Funding	Countries	Number of trainees
1. VU-CIDRZ AITRP D43TW001035 (18 years + consistent funding)	NIH/FIC	Multiple, including Nigeria	93 (masters/doctoral), 300 in-country short course trainees
2. Int. Clinical Res. Scholars and Fellows Support Center (R24TW009337)	NIH/FIC	Multiple	536 fellows
3. Vanderbilt-Emory Cornell-Duke Consortium for Global Health Fellows (R25TW009337)	NIH/FIC	Multiple—14 countries	88 fellows
4. Vanderbilt-Emory Cornell-Duke Consortium for Global Health Fellows (D43TW009337)	NIH/FIC	Multiple, including Nigeria	55 fellows
5. Vanderbilt-Zambia Network for Innovation in Global Health Technologies (D43TW009348)	NIH/FIC	Zambia	17 fellows
6. University of Guyana MPH Programme	CDC	Guyana	45 masters students
7. VU-Mozambique Collaborative Research Ethics Education Program (FoCEP, R25TW009722)	NIH/FIC	Mozambique	2 masters, 151 in-country short course trainees
8. UEM Partnership for Research in Implementation Science Mozambique (PRISM, D43TW009745)	NIH/FIC	Mozambique	4 masters students, 75 in-country short course trainees
9. UNZA-Vanderbilt Partnership for HIV-Nutritional Research Training (UVP, D43TW009744)	NIH/FIC	Zambia	11 PhD students, 200 in-country short course trainees
10. Partnership for Research in Emerging Viral Infections-Sierra Leone (PREVSL, D71TW010411)	NIH/FIC	Sierra Leone	N/A
11. Vanderbilt Institute for Research Development and Ethics (VIRDE)	VIGH funds	Multiple	48 short course trainees
TOTAL			155 Masters/doctoral degrees, 696 fellows, 774 short-course trainees

*VU* Vanderbilt University, *CIDRZ* Center for Infectious Disease Research in Zambia, *AITRP* AIDS International Training and Research Program, *MPH* Masters in Public Health, *UEM* University Eduardo Mondlane, *UNZA* University of Zambia School of Medicine, *NIH* National Institutes of Health, *CDC* Centers for Disease Control and Prevention

## Programme administration

The V-BRCH programme leadership includes an Executive Committee of six senior Vanderbilt and AKTH faculty. The Executive Committee is assisted by a multidisciplinary team of AKTH and Vanderbilt faculty mentors. These mentors include experts in HIV, NCDs, clinical trials, biostatistics, implementation science, and health informatics. A Training Advisory Committee (TAC) of six members includes leaders with expertise in global health research training and Nigeria-specific work history. The VIGH Office for Education and Training will supervise and coordinate administrative management of V-BRCH with programme staff at AKTH.

## Specific aims

*Specific aim 1:* Solidify an atmosphere of continuous mentoring and skills acquisition for AKTH physician faculty in conducting clinical trials in HIV-associated NCDs via short- and medium-term learning opportunities, paired mentoring arrangements and seed research funds to eligible mentor–mentee teams.

### Local (Nigeria) workshops

We will convene two 2-week, intensive, on-site workshops annually in Kano, Nigeria for physicians drawn from the infectious diseases, cardiology, nephrology, gastroenterology, neurology, paediatrics, pathology, and public health divisions at AKTH. These workshops will facilitate the acquisition of multidisciplinary research skills and offer opportunities for professional development to support broad-based investigations of HIV-associated NCDs. They will focus on the foundation, methodologies, and application of patient-oriented clinical trial research and will cover pertinent topics such as the epidemiology of HIV-associated NCDs, development of informed consent documents, data collection and management, data integrity, protocol development and deviations, and research ethics. The workshops will also cover mentorship and leadership training, evidence synthesis (e.g., systematic reviews), qualitative research methods, stakeholder engagement, knowledge translation (dissemination), and grant writing. The on-site workshops will also serve as an avenue for one-on-one mentoring between United States-based mentors and local team members.

### Vanderbilt Institute for Research Development and Ethics (VIRDE)

We will select three junior faculty members per year from the cohort of workshop trainees to attend the annual 1-month-long VIRDE programme in Nashville. VIRDE is intended to equip trainees with the skills necessary to conduct responsible human subjects research and develop grant proposals. Trainees are matched with Vanderbilt faculty mentors who shepherd them through the development of hypothesis-driven research grant applications, many of whom will continue to provide one-on-one training after VIRDE training is complete. VIRDE trainees will also complete 12 contact hours of tailored coursework in research ethics and integrity. Toward sustainability, VIRDE training for V-BRCH trainees will be moved to Nigeria and conducted as Vanderbilt/AKTH–co-led training in year 4 and a fully AKTH-led training in year 5.

### Faculty development fellowships

This medium-term training will comprise 3-month-long fellowships in Nashville for research-oriented AKTH physician faculty (assistant professor level) for focused training in key areas of clinical trials implementation.

Two faculty trainees will be identified and recruited in each of four years. Selection will be conducted by the Executive Committee based on an assessment of each fellow’s needs, strengths, and interests, and the recommendations of the TAC. Priority will be given to junior faculty from the AKTH divisions listed above and who serve as co-investigators on ongoing NIH-funded studies. A Vanderbilt mentor with NIH funding experience will be identified for each trainee and will work with them to create an individual development plan (IDP), outlining learning competencies, recommended coursework, timeline, and objective criteria for determining success.

Throughout the fellowship, trainees will shadow established clinical trialists at Vanderbilt, sharing best practices and lessons learned with each other through peer mentoring panels, regular presentations, and monthly career development seminars. Training will be tailored to the individual faculty fellow through two courses:

*Clinical and translational scientist short course at Vanderbilt (8 weeks):* This course is modelled on Vanderbilt’s Physician Scientist Development programme [24]. Fellows in this course co-mentor each other through a mentoring panel, conduct regular Work-in-Progress presentations, and participate in twice-monthly career development seminars and case studies on responsible conduct of research (RCR) and research administration. Fellows will have access to Vanderbilt’s library resources, biostatistics consultations, manuscript preparation work groups, and design studios that bring together methodology experts to vet research proposals. These resources will help fellows generate new hypotheses, enhance the quality of their proposals and publications, and improve their chances of funding success.

*Implementation science and quality improvement short course (4 weeks):* This is an intensive curriculum devoted to building implementation research skills by introducing trainees to research concepts and methods applicable to health service delivery, to include: patient-reported data and outcomes, comparative effectiveness research, pragmatic trials, and implementation science basics. Fellows will participate in didactic training and workshops at Vanderbilt’s Center for Clinical Quality and Implementation Research. Fellows will also participate in biweekly scholarly series, journal clubs, and a peer mentorship group, and will audit graduate courses within the MPH programme.

### Seed awards for pilot studies

We will pair fellows with V-BRCH mentors with NIH-funding experience based on a pre-fellowship survey for continuous mentoring. Mentor–mentee teams will compete for seed research funding (two teams awarded per year) to conduct local HIV-associated NCD studies and generate pilot data. The research projects must be directly relevant to HIV-NCD priorities at AKTH. An example of this might be implementation of a pilot trial to generate preliminary data to support a full-scale NIH R-series clinical trial.

*Specific aim 2:* Create the next generation of highly skilled physician public health leaders through long-term (master’s degree level) training in clinical trial research methodology.

Long-term training opportunities consist of a 2-year Master of Public Health (MPH) programme at Bayero University Kano (for eight Nigerian trainees) and a 2-year MSCI at Vanderbilt (for two Nigerian trainees). The candidate pool will comprise physicians from the AKTH divisions listed above. We will prioritize junior physician faculty (within 3 years of completing training) who have demonstrated interest in clinical research, as evidenced by involvement in ongoing clinical studies and publications on relevant HIV-associated NCD topics.

### MPH programme at BUK

The MPH programme at BUK requires students to complete a total of 38 credits of coursework that comprise 22 core credits and 8 elective credits. Students are also required to complete 4 weeks of a supervised practicum, field placement in the student’s specialty, or research project (2 credits) and complete a dissertation (6 credits). Additional curriculum refinement will also be conducted, as advanced clinical trials methodology is currently not adequately covered in the MPH programme.

### MSCI programme at Vanderbilt

This programme includes 21 months of coursework in Nashville and 3 months in the field in Nigeria and provides trainees with a strong foundation in the core skills and methods of clinical and translational research. Components of the programme include a mentored research apprenticeship that pairs the trainee with an established physician-scientist with experience in clinical and translational research; didactic work (35 credit hours in study design, biostatistics, ethics, drug development, and data analysis); monthly clinical scientist career seminars and a final project (first-authored manuscript to a peer-reviewed journal, completed grant proposal, or master’s thesis).

*Specific aim 3:* Build institutional capacity in HIV-associated NCD clinical trial research via short-term regional courses, staff development workshops, and MPH curriculum refinement.

### South-South collaborative training

V-BRCH will leverage seminars, workshops, and other activities conducted by NIH-funded research training programmes in Nigeria and other African countries (South-South collaborative opportunities) to take advantage of complementary training, peer learning and research networking opportunities. Each year, we will target up to four AKTH faculty and research staff already involved in research at AKTH, prioritizing those with established work relationships with NIH-funded, HIV-related research at AKTH. These activities will facilitate continuous mentoring and enhance intra-regional research collaboration.

### Staff research development fellowships

We will support annual 2-week trainings in Nashville for AKTH research staff (coordinators, research assistants/associates) involved in clinical trial administration and regulatory oversight (two staff members every year for 4 years). Staff trainees will regularly review and discuss case studies on RCR and research administration. The goal is to strengthen the capacity of AKTH in research ethics, governance, and administration, with specific training to include:

1. *Best practices in clinical trial administration, regulatory review, and RCR:* Staff trainees will attend a 1-week RCR programme with Vanderbilt’s Biomedical Research Education and Training division. In addition, trainees will observe four meetings of the Vanderbilt Institutional Review Board (IRB). Trainees will be required to complete the Food and Drug Administration (FDA)’s online course on regulatory review and research, and human subjects and good clinical practice training through the Collaborative Institutional Training Initiative (CITI) programme.
2. *Improving efficiency in administrative and technical management of funded clinical trials:* Trainees will be taught to use REDCap, a research data collection and management software platform that has been adopted by some 1660 institutions in 94 countries [25]. Using REDCap, a single portal can be created to track grant submissions and run an operational database to track progress of ongoing clinical trials. As the 2-week Nashville visit does not provide sufficient time for this purpose, we will enrol trainees in the popular Coursera course “Data Management for Clinical Research” taught by a Vanderbilt faculty member.
3. *Sharing experiences *via* in-person meetings and site visits:* Vanderbilt’s Trial Innovation Center (TIC) studies how clinical trials of therapies can be conducted more rapidly and efficiently. Trainees will meet with TIC leadership and the Study Start-up Core, whose mandate is the design and implementation of practices to get trials underway as quickly as possible. Trainees will also meet with Vanderbilt Clinical Trials Center staff to observe best practices in the review, preparation, and conduct of clinical trials. Finally, trainees will meet with leading Vanderbilt clinical trialists to learn first-hand about their experiences in conducting clinical trials and exchange ideas. The emphasis of discussions at these meetings will be on logistical considerations, study start-up, and research compliance and administration.

### MPH curriculum refinement

We will revamp the MPH curriculum at AKTH in consultation with our AKTH collaborators by developing advanced modules in core quantitative and methodological principles of clinical trials. The modules will be based on Vanderbilt’s MSCI curriculum and will include topics such as randomization, ethical considerations for placebo-controlled studies, regulatory issues, study monitoring, specimen integrity, protocol development, and stakeholder roles and responsibilities. We will include integrated scenarios, case studies based on real-life experiences, and lectures from subject matter experts. The new courses will be taught by visiting Vanderbilt faculty in the first 2 years and will then be transitioned to AKTH faculty to ensure sustainability.

## Trainee recruitment and selection

The candidate pool for this fellowship programme will include junior faculty and staff members from AKTH. BUK graduates over 100 physicians every year. The MPH programme at BUK has an annual intake of 10–15 trainees. The MPH/MSCI trainees will be drawn from a pool of newly minted attending physicians (3 years or less post-graduation) at AKTH who have demonstrated interest in HIV-associated NCD research. Staff trainees will include research assistants, associates, and coordinators currently involved in ongoing clinical research projects at AKTH.

We will recruit the most promising candidates who are interested in clinical trial research and are likely to contribute to the development of NCD-HIV clinical trials in Nigeria but who lack the appropriate research skills. We will identify a total of 10 Nigerian physician scientists for MPH or MSCI training. Selection for V-BRCH support for the MPH/MSCI will be based on:

- Admission to the BUK MPH or Vanderbilt MSCI programmes, based on specific programme requirements.
- Physicians with demonstrated interest in research in NCD comorbidities of HIV, with evidence of commitment to a career in academic research. We will target students wishing to develop a spectrum of research relevant to HIV/NCD clinical trials.
- Current employment with AKTH (preferably < 3 years). This will allow V-BRCH to optimize the likelihood of long-term retention of graduates as faculty researchers.

Recruitment of trainees will include referrals from faculty and senior researchers at AKTH. Qualified candidates may also make self-referrals. We will advertise all training opportunities through AKTH and Vanderbilt, on our V-BRCH website, and by word of mouth through our investigators. Preference will be given to candidates with some prior research experience and who currently work with or have links with established Vanderbilt-affiliated research programmes at AKTH.

Applicants for MPH/MSCI training will be required to submit the following materials: (1) curriculum vitae (CV), (2) personal statement, (3) transcripts, (4) recommendation letters, (5) brief hypothesis-driven research concept proposal, and (6) concise training plan. MSCI candidates must also meet Vanderbilt admission requirements, including evidence of fluency in English and passing Graduate Record Examinations (GRE) scores. Candidates will be interviewed by AKTH and Vanderbilt University Medical Center (VUMC) investigators.

For medium-term training (faculty fellowships), we will use a similar application process as that described above, with the exception of the research concept proposal. All fellowship applications will be reviewed by the TAC for final selection and approval. For workshop applicants, we will require only a CV, a brief personal statement about their research and career interests, and a reference letter. We will encourage and support applications from qualified women, targeting at least 50% female representation across all V-BRCH training opportunities.

## Mentorship approach

Longitudinal mentoring experiences for trainees will be tightly linked with priority HIV-NCD research needs identified by AKTH. By taking this approach, the research studies proposed by each trainee will have direct policy relevance that will increase the likelihood of sustainable, national impact. Each medium-term trainee (faculty fellow) or long-term trainee (MPH/MSCI student) will be co-mentored by an AKTH faculty mentor and a Vanderbilt faculty mentor. Dual mentorship will ensure that each trainee receives the full benefit of the expertise, perspective, and attention of faculty mentors. Trainees will be paired with mentors based on similar research interests, skill sets, and personalities. On a case-by-case basis, we will assign additional mentors to help trainees address specific content areas or career planning.

V-BRCH trainees will be required to develop an independent development plan (IDP) in which they lay out their expectations and long-term goals. Mentors will review the IDPs with their mentees each year. Trainees and mentors will also submit regular progress reports to the Executive Committee, detailing trainee progress and providing opportunities for mentors and mentees to reflect on their interactions. Each mentor–mentee team will be required to meet either in person or online at least monthly to ensure continuous and supportive collaborative communication. Using the mentee-driven model of mentorship, trainees will learn to assume active roles in the mentorship process, including setting agendas for mentoring meetings and emailing summaries of key points and action items to the mentors after each meeting. Mentors will be encouraged to access mentoring resources at VUMC and AKTH and online.

## Programme evaluation

**Fig. 1:**
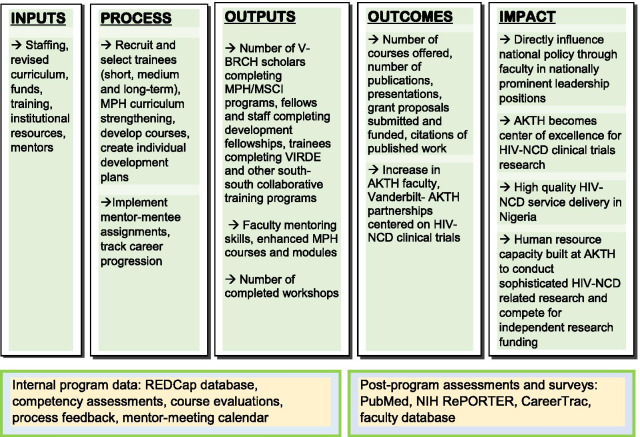
V-BRCH programme inputs, processes, outputs, outcomes, and impact

The success of V-BRCH will be based on objective measures and a mixed methods approach to evaluation. The evaluation logic model (Fig. 1) includes the following elements: inputs (resources), processes (programme components), outputs (products of programme implementation), outcomes (changes in trainees’ knowledge, skills, attitudes, practices), and impact (trainee career trajectory, sustainable changes in health conditions). The REDCap software programme will be used to track, evaluate, and report short- and long-term training outputs, outcomes, and impact. All evaluation information will feed back to the TAC and Executive Committee to enhance programme effectiveness.

Baseline evaluation data will be collected on all new trainees upon programme entry. The data will include trainee demographics, prior training, research experience, and scholarly accomplishments. In addition to the IDPs, trainees will chronicle their research and research training activities and accomplishments via an online REDCap survey twice annually. The survey will include courses taken, the quality of mentoring, satisfaction with mentoring, research and ethics training, and other training activities. V-BRCH mentors will also evaluate their mentees every 6 months with regard to mentee progress, attainment of IDP goals, scholarly productivity, communication, professionalism, career motivation, and comments on areas of improvement, among others. The Executive Committee will review these surveys, and any problem areas will be identified and addressed.

Trainees and alumni will be surveyed annually via REDCap to assess the programme’s progress. Metrics will include demographics; position, institution, and rank; publications; grants submitted and awarded; presentations at scientific conferences; new research partners and collaborations; satisfaction with mentor relationships and new academic appointments, especially in HIV-NCD research. Alumni surveys will also assess the overall quality of the programme, career accomplishments, and whether certain programme elements should be added or emphasized in greater depth.

*Other evaluations:* After each training activity (workshops, VIRDE, other short courses), trainees will complete an evaluation of the activity via an online survey that includes an assessment of course learning objectives, quality of instruction, areas for improvement and unmet needs, and relevance to HIV-NCD research. Based on this feedback, course content and structure will be modified, if necessary. All MPH/MSCI trainees will also participate in exit interviews upon programme completion. The interviews will cover trainee experience, satisfaction, perception of quality of training, and immediate and long-term career plans. Annual mentor surveys will solicit feedback from mentors regarding their experiences, satisfaction with the process, and recommendations for programme improvement.

The individual-level information obtained from the evaluation surveys will be aggregated annually to produce cumulative metrics by category. All cumulative data will be entered into NIH’s CareerTrac database. Qualitative data will be triangulated with survey data to obtain a comprehensive analysis of programme impact. The TAC will formally assess the impact of the V-BRCH programme annually and make recommendations to the Executive Committee. Evaluation data will be shared with AKTH leadership to enable strategic planning and policy, as well as to facilitate sustainability.

## Progress to date

Two Executive Committee meetings were held in April/May 2020, and the first TAC meeting is scheduled for July 2020. The first workshop in Nigeria is scheduled for November 2020. The BUK/AKTH MPH curriculum revision to incorporate advanced clinical trial courses is ongoing. The 2020 VIRDE workshop has been cancelled because of COVID-19 restrictions; the first VIRDE workshop will therefore be held in Fall 2021. Preliminary discussions with partners regarding scheduling of South-South enrichment training opportunities are ongoing.

## Anticipated challenges and sustainability

The proposed programme is not without its challenges. Nigeria is a complex environment with its attendant security, administrative, and socioeconomic problems. Frequent industrial strikes by university lecturers could impact our ability to ensure that new courses developed from the revised MPH curriculum are taught in a timely manner. Security issues related to terrorism in the northeast and highway crimes could affect travel plans. Fortunately, Kano has been spared major incidents so far. The COVID-19 pandemic has also impacted travel and academic calendars, including the training programmes planned for Nashville. We have had to adjust the training calendar to accommodate those disruptions.

There is a need to ensure that resources and investments required for the programme to be successful and sustainable are identified. We have specific plans for sustainability; for example, we will transition new courses developed from the revised MPH curriculum from Vanderbilt faculty to AKTH faculty, move VIRDE training to Nigeria as a fully AKTH-led training in year 5, and share programme evaluation data with AKTH leadership to enable strategic planning. Our trainees are all AKTH employees; hence the skills, knowledge, and competence they acquire can be readily cascaded down to their mentees. We will share leadership and responsibility for the programme with our AKTH partners, and will support their future applications for funding to implement similar initiatives.

Many capacity-building initiatives have traditionally been focused on individuals. Such interventions which increase the skills base of individuals and the resources of organizations are necessary but not sufficient for lasting change to occur. It is also necessary to stimulate the development of an environment that rewards and values research and that inspires an atmosphere in which evidence is used to create national policy. Future programmes should aim to build capacity beyond the technical skills necessary to conduct quality research, to cover the full research cycle from fundraising and project planning through research design and delivery, to dissemination and policy engagement.

## Conclusion

The conduct of clinical trials in LMICs is hindered by significant challenges, making these settings unattractive to researchers from resource-replete countries. Ethicists advocate that global health research should be integrated with capacity-building strategies that are responsive to local needs and add value to local conduct of research [26, 27]. We propose a coordinated approach to build the capacity of AKTH investigators to successfully initiate and implement HIV-NCD clinical trials. Vanderbilt’s extensive research investment in AKTH strengthens our ability to reinforce local capacity to conduct rigorous, impactful, and responsive clinical trial research. We include a thoughtful transition plan that will empower Nigerians to progressively assume training leadership roles. V-BRCH will create a cohort of skilled Nigerian scientists able to design and implement high-quality research that will generate evidence to inform policy and practice and eventually lead to improved outcomes for Nigerians impacted by HIV-associated NCDs.

## Data Availability

Not applicable.

## Abbreviations

AKTH: Aminu Kano Teaching Hospital
CITI: Collaborative Institutional Training Initiative
CV: Curriculum vitae
FDA: Food and Drug Administration
GRE: Graduate Record Examinations
IRB: Institutional review board
MPH: Masters in Public Health
MSCI: Master of Science in Clinical Investigation
NCDs: Noncommunicable diseases
RCR: Responsible Conduct of Research
TAC: Training Advisory Committee
V-BRCH: Vanderbilt Building Research Capacity in HIV and Non-communicable Diseases
VUMC: Vanderbilt University Medical Center
